# Student engagement assessment using multimodal deep learning

**DOI:** 10.1371/journal.pone.0325377

**Published:** 2025-06-10

**Authors:** Lijuan Yan, Xiaotao Wu, Yi Wang

**Affiliations:** College of Mathematics and Statistics, Huanggang Normal University, Huanggang, Hubei, China; Zhejiang Normal University, CHINA

## Abstract

Student engagement assessment plays an important role in enhancing students’ positive performance and optimizing teaching methods. In this paper, a multimodal deep learning framework is proposed for student engagement assessment. Based on this framework, we propose a method for engagement assessment that utilizes data from three modalities: video, text, and logs. This method implements the extraction of engagement indicators, the fusion of asynchronous data, the use of deep learning models to evaluate engagement levels, and the use of gradient magnitude mapping to further distinguish subtle differences between engagement levels. In subsequent empirical studies, we explore the applicability of several popular deep CNN models in this method and validate the reliability of the engagement quantification results using statistical methods. The analysis results demonstrate that the framework, which combines multimodal asynchronous data fusion and deep learning models to assess engagement, is both effective and practical.

## 1 Introduction

Over the past few decades, researchers and educators have shown a growing interest in student engagement. Student engagement is a crucial factor in improving learning outcomes [1], creating a positive learning environment [2], and enhancing the overall quality of education [3]. Assessing student engagement helps identify areas where students may be disengaged or struggling. It facilitates personalized learning experiences. By understanding student engagement, educators can adapt their instruction to meet the unique needs of each students, ensuring that their individual learning styles and preferences are considered. It also contributes to educational research and the development of innovative teaching methods and technologies, which can benefit students in the long run.

With the increasing proliferation of educational applications (e.g., adaptive learning system [4], educational robot [5] and intelligent assisted teaching platform [6]), the need for accurate engagement assessment has also become increasingly urgent. In recent years, various data mining approaches have been introduced into the assessment process and have yielded significant results [7,8]. By applying clustering algorithms such as k-means, researchers have identified different levels of student engagement patterns in online and blended learning environments [9,10]. By utilizing classification algorithms such as decision tree, random forest and logistic regression, researchers have achieved high accuracy in predicting student engagement levels, thereby improving dataset quality and enabling more effective interventions and decision-making in educational institutions [11]. Compared to traditional methods such as self-report measures [12–14], experience sampling techniques [15,16], teacher ratings [17], and observations [18–20], these approaches can automatically analyze data without frequent disruptions to students, leading to more objective outcomes.

In the educational context, student engagement assessment (SEA) data possesses unique characteristics. It is dynamic, typically collected from students’ learning processes, reflecting the constantly evolving levels of engagement during learning activities. Hence, SEA data exhibits temporal patterns, including fluctuations in engagement levels over time and periodic changes related to course activities. SEA data often comprises multiple modalities, such as interactive texts, affective attitudes, behavior logs, etc. These diverse data sources provide a more comprehensive understanding of student engagement but also increase the complexity of data analysis and interpretation. SEA data presents unique challenges and opportunities, requiring specialized methodologies for effective analysis and application in educational settings.

Multimodal deep learning is an emerging and powerful data mining technique that combines information from multiple data modalities, enabling more accurate and comprehensive analysis and predictions. It has achieved outstanding results in fields such as healthcare [21], transportation [22], and finance [23]. However, its application in the field of education is still in its stages, and several challenges remain that require further exploration. Among these, data fusion and interpretability stand out as significant hurdles. Combining and processing data from multiple modalities can be complex and may necessitate the development of specialized techniques. Combining information from different modalities is a prerequisite for obtaining meaningful predictions. Ensuring seamless integration and handling heterogeneity is an ongoing challenge. Explaining the decisions made by multimodal models and understanding their internal workings is crucial to make the results of educational data analysis more practically valuable. Applying the model’s interpretability to SEA is an ongoing research area.

To address these challenges, we propose a multimodal deep learning framework for SEA. Building upon this framework, we propose a method for assessing engagement using three modalities: video, text, and log data. This method extracts learning engagement information from multiple modalities, employs deep learning models to evaluate engagement levels, and utilizes model interpretability to distinguish differences between these levels.

Our major contributions are summarized as follows:

A general method for learning engagement data fusion is proposed, utilizing multivariate time series to unify and represent complementary feature information from different modalities, thereby providing a more accurate depiction of students’ learning engagement.

For coarse-grained assessment of student engagement, we employ time series deep learning algorithms. On a real education dataset, we explore the applicability of four mature CNN models for identifying levels of student engagement.

For fine-grained assessment of student engagement, we propose a quantification algorithm for engagement scores. We utilize gradient magnitude mapping and statistical methods to quantify the contribution of each data feature to the final classification result through reverse computation.

The remainder of this paper is structured as follows: Sect 2 surveys the state-of-the-art on related multimodal deep learning. Sect 3 presents a general framework and a specific method for assessment using three modalities. Sect 4 discusses the exhaustive experimental evaluation method which was applied to evaluate the effectiveness and reasonableness of our framework. Finally, Sect 5 summarizes the key findings of the presented work and highlights major directions for future research.

## 2 Related work

In this section, we give a review of background work related to multimodal deep learning.

Modality are channels of information, or anything that communicates meaning in some way, including sounds, videos, words, gestures, eye movements, facial expressions, physiological signals, etc. Multimodal data combines at least two modalities. A key property of multimodality is complementarity, in the sense that each modality brings to the whole some type of added value that cannot be deduced or obtained from any of the other modalities in the setup [24].

Recent advances in deep learning have led to significant progress in multimodal data analysis. At present, some researchers have used multimodal deep learning models for SEA and got the state-of-the art results. Chen *et al*. [25] integrated the students’ eye movement data, facial expression data, and EEG data, and used a multi-layer convolutional neural network (CNN) for analysis to obtain information such as students’ cognitive engagement. Li *et al*. [26] assessed students’ cognitive engagement using multimodal data including EEG data, eye movement data, heart rate data, and galvanic skin response data. They adopted a deep learning algorithm based on a multi-layer attention mechanism to combine different data modalities to obtain more accurate evaluation results. Huttunen *et al*. [27] integrated the EEG data of the students and facial expression data and analyzed them using a multilayer perceptron (MLP)-based neural network to obtain student engagement information.

Current studies have shown that using multimodal deep learning can provide a more comprehensive and accurate assessment of student engagement. For example, Monkaresi *et al*. [28] use two modalities, video-based estimation of facial expressions and heart rate, to improve the accuracy of engagement recognition in a structured writing activity. Psaltis *et al*. [29] apply body motion and facial expression analysis to identify students’ cognitive and behavioral engagement in a game. The proposed multimodal affective recognition algorithm improves classification accuracy. Multimodal deep learning can help understand students’ learning status and needs from multiple perspectives, providing valuable information for educators and researchers to develop better teaching strategies and improve learning outcomes. In this paper, we leverage multimodal deep learning to recognize student engagement level, and also leverage its interpretability to distinguish subtle differences among students at the same engagement level.

## 3 Methods

This work has not required ethical approval as it is based on information collected as part of a routine teaching quality assessment. The volunteer participants were aged between 19 and 20, and all participants were informed in writing of the study’s objectives, and their consent was obtained for the connection of learning process data. All the collected data has undergone anonymization.

### 3.1 Framework for multimodal engagement assessment

The key to multimodal learning engagement assessment lies in creating a shared representation space, which more accurately describes students’ learning engagement by effectively capturing complementary information from different modalities. Currently, there are many well-established multimodal data analysis frameworks for engagement assessment. For example, D’Mello *et al*. proposed the Advanced, Analytic, Automated (AAA) measurement [30]. Yu *et al*. introduced the LEARNSense framework [31]. These studies discuss the strengths and weaknesses of using multimodal data, such as behavioral and physiological data, to measure engagement. Building on these works, this paper proposes a new framework. This study proposes a deep learning-based assessment framework for asynchronous fusion of multimodal data. The framework consists of the following three stages.

(1) Engagement Indicator Extraction Stage

In this stage, engagement indicators are extracted from data of various modalities, such as images, audio, and text. Typically, these data are processed separately. Each modality has its own encoder, which transforms the input data into a set of feature vectors that capture the engagement information from the modality data. Common methods for extracting engagement indicators from modality data are listed in Table 1.

**Table 1:** Methods for Extracting Engagement Indicators from Multimodal Data.

Modality	Engagement Indicator	Extraction Methods
Text	Behavioral Engagement	- Term Frequency (TF)eak - Term Frequency-Inverse Document Frequency (TF-IDF)eak - N-gram Models
	Emotional Engagement	- Sentiment Lexicon Matching (e.g., LIWC, SentiWordNet)eak - Machine Learning-based Sentiment Analysis (e.g., SVM, LSTM)
	Cognitive Engagement	- Topic Modeling (e.g., LDA, Latent Dirichlet Allocation)eak - Keyword Extraction (e.g., RAKE, TextRank)
Video	Behavioral Engagement	- Action Recognition (e.g., OpenPose, MediaPipe)eak - Head Pose Estimationeak - Gesture Detection
	Emotional Engagement	- Facial Expression Recognition (e.g., FACS, Facial Action Coding System)eak - Deep Learning-based Emotion Classification
	Cognitive Engagement	- Eye Tracking (e.g., Fixation Analysis, Scanpath)eak - Attention Detection (e.g., Head Orientation, Gaze Direction)
Logs	Behavioral Engagement	- Clickstream Analysis (e.g., Click Frequency, Dwell Time)eak - Interaction Event Statistics (e.g., Mouse Movements, Scrolling Behavior)
	Emotional Engagement	- Sentiment Labels (e.g., User Feedback Ratings)eak - Log-based Emotion Inference (e.g., Interaction Pattern Analysis)
	Cognitive Engagement	- Task Completion Analysis (e.g., Task Success Rate, Error Rate)eak - Learning Curve Analysis (e.g., Knowledge Mastery Progress)
Audio	Behavioral Engagement	- Voice Activity Detection (VAD)eak - Speech Duration Statistics
	Emotional Engagement	- Speech Emotion Analysis (e.g., Pitch, Speech Rate, Energy Features)eak - Machine Learning-based Emotion Classification
	Cognitive Engagement	- Speech Content Analysis (e.g., Keyword Extraction, Semantic Analysis)eak - Speech Pause and Filler Word Analysis
Physiological Signals	Behavioral Engagement	- Heart Rate Variability (HRV) Analysiseak - Galvanic Skin Response (GSR) Detection
	Emotional Engagement	- Electroencephalogram (EEG) Emotion Classificationeak - Facial Electromyography (EMG) Analysis
	Cognitive Engagement	- Electroencephalogram (EEG) Attention Detectioneak - Functional Near-Infrared Spectroscopy (fNIRS) Cognitive Load Analysis

(2) Data Fusion Stage

After extracting feature vectors of engagement indicators from different modalities, aligning the data on the timeline preserves temporal characteristics and the relationships between modalities. Determining how to align data on the timeline is crucial. This may require the use of timestamps, sampling rates, or event markers to synchronize data from different modalities. Temporal alignment can be achieved using the following methods:

Interpolation: Using interpolation methods to resample data to the same time points.

Timestamp Matching: Matching timestamps of different modality data to enable comparison at the same time points.

Event Alignment: Using event markers to align data, such as aligning all modality data to the occurrence of a specific event.

The time-aligned data is then fused into multivariate time series. This may involve stacking, concatenating, or combining data from different modalities into a single multivariate data point. Data fusion may also require normalization and standardization to ensure that data from different modalities are on the same scale.

(3) Engagement Assessment Stage

The engagement assessment using deep learning leverages the joint representation generated in the time alignment stage to make predictions or decisions. Various deep learning algorithms suitable for multivariate time series can be employed to process the fused data described above. The specific methods used in the assessment stage may vary depending on the quantification task and the type of data being processed. For example, in learning engagement pattern recognition tasks, deep learning classification algorithms output probabilities of different engagement levels.

This framework provides a comprehensive approach to assessing learning engagement by integrating multimodal data through asynchronous fusion and deep learning techniques.

### 3.2 Empirical methodology for three modalities

**Fig 1:**
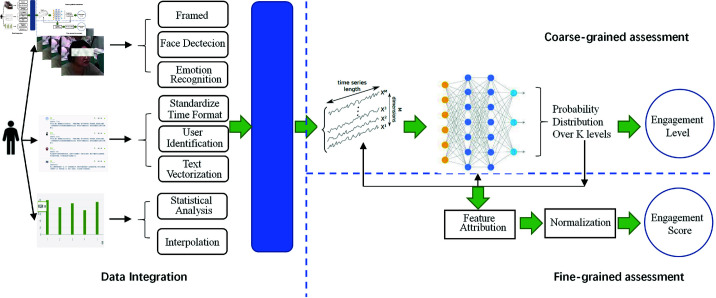
A multimodal deep learning method for engagement assessment.

Based on the above framework, we conduct an empirical study on engagement assessment, which utilized data from three modalities, video, text and log. Empirical methodology in this work is shown in Fig 1. It mainly consists of three parts: engagement indicator extraction, data fusion, coarse-grained assessment based on deep learning models, and fine-grained assessment based on model interpretability.

#### 3.2.1 Engagement indicator extraction.

Video data can be recorded during the learning process. Employing emotion recognition allows us to understand the temporal fluctuations in the student’s emotional engagement status [32,33]. Video is framed into sequence of image frames $V_{i}=\left(frame_{i1},frame_{i2},\ldots ,frame_{in}\right)$ at a certain time intervals, and the face region is extracted on each image frame and then aligned according to the timestamp. $Face_{i}=\left(Face_{i1},Face_{i2},\ldots ,Face_{in}\right)$ denotes a face images sequence of the *i*_th student sample. Using facial expression recognition technology, face images can be further encoded into emotional state label. In this paper, we train a Convolutional Neural Network (CNN) model using the open-source facial image dataset fer2013 [34] for image classification. The face images are divided into 7 basic emotion categories, corresponding to numeric state labels 0-6. The specific mapping between numeric labels and emotion categories is as follows: 0: Anger 1: Disgust 2: Fear 3: Happy 4: Sadness 5: Surprise 6: Neutral.

So a discrete real-valued sequence $E_{i}=\left(E_{i1},E_{i2},\ldots ,E_{in}\right)$ detected from sequence $V_{i}$, which can reflect how the i_th student’s emotional state has changed over time.

A lot of text data is produced when students communicate with each other via learning platforms or instant messaging applications. We can gauge how well students are learning and completing assignments by analyzing interactive content, which also serves as a reflection of the students’ cognitive engagement [35,36]. The text data preprocessing steps can be carried out as follows: first, the collected text chat data need to be converted into standardized time format and irrelevant information removed. Next, identify different students in the text and assign a unique identifier to each. Split the text data into subsets based on student identifiers. And finally, Vectorising the text data for each student. TF-IDF (Term Frequency-Inverse Document Frequency) is a common method of text vectorization. In this work, we calculate the sum of the TF-IDF values of each student’s published words within a certain time interval.

For the interactive text document *d*, the student *i* expresses text messages containing *n* words within a specific period of time *t*, then the TF-IDF value of the word *j* can be calculated.

Among them, TF represents the frequency of words in the document. Therefore, the frequency of word *j* expressed by the student *i* in document d can be represented as Eq 1.

$$TF_{i,j}=\frac{n_{j,d}}{\sum_{k}n_{k,d}}$$ (1)

where, *n*_*j*,*d*_ represent the number of times the word *j* appears in document *d*.

IDF (Inverse Document Frequency) represents the uniqueness or rarity of a word in the entire document collection. It is typically calculated as the logarithm of the total number of documents divided by the number of documents that contain the word. The IDF for the word *j* can be represented as Eq 2.

$$IDF_{j}=1+log\frac{\left|D\right|}{df_{j}}$$ (2)

where, *df*_*j*_ is the number of messages that contain word *j*. |D| is the total number of messages in document *d*. The TF-IDF value is obtained by multiplying the TF and IDF scores. This value represents the importance of a word in a specific document, illustrated in Eq 3.

$${\text{TF-IDF}}_{i,j}=tf_{i,j}\times idf_{j}$$ (3)

The sum of TF-IDF value for the word expressed by student *i* within a specific period of time *t* can be represented as Eq 4.

$${\text{TF-IDF}}_{sum}=\sum_{j=1}^{n}{\text{TF-IDF}}_{i,j}$$ (4)

So, the raw interactive text data is also encoded into a discrete real-valued sequence $T_{i}=\left({\text{TF-IDF}}_{sum1},{\text{TF-IDF}}_{sum2},\ldots ,{\text{TF-IDF}}_{sumn}\right)$ . Encoded data can reflect student’s acquisition and understanding of new information during the communication process, which denote changes in student’s cognitive states over time.

The log data can be obtained from the application, which to some extent can reflect the students’ behavioral engagement [37,38].The log data record various activities of students in application, including login, course enrollment, video playback, quiz attempts, assignment submissions, course completions, and logout. These log data can be utilized to analyze user behavior, course participation, learning progress, and other relevant metrics and insights. Parsing the raw log data can extract relevant information, such as timestamp, log level, message, and other relevant attributes. By performing statistical analysis, interpolation, and other processing steps, log data can be transformed into a meaningful time format suitable for further analysis, visualization, and interpretation.

In this work, we extracted the student’s in-class quiz scores, along with the timestamps of when the quiz was started and submitted. The scores correspond to the timestamps when the quizzes were completed. So, the real-valued data on the timeline is too sparse. We use linear interpolation within the quiz start and submit time interval to create a more continuous representation of the data. Let’s denote the time series of quiz scores as *S*_*i*_, where i represents the *i*_th student. We assume a linear relationship between adjacent data points. For each missing timestamp within the interval between the quiz start time(*t*_*s*_)and the end time(*t*_*e*_), we calculate the interpolated quiz score using linear interpolation, illustrated in Eq 5.

$$Q_{m}=Q_{t_{s}}+\frac{Q_{t_{e}}-Q_{t_{s}}}{t_{e}-t_{s}}\times \left(t_{m}-t_{s}\right)$$ (5)

where, the value of $Q_{t_{s}}$ is zero, the value of $Q_{t_{e}}$ is the student’s in-class quiz score. Finally, $Q_{i}=\left(Q_{m1},Q_{m2},\ldots ,Q_{mn}\right)$ denotes changes in student’s behavioral states on quizzes over time.

#### 3.2.2 Data fusion.

When the same time interval is used, these modal data can be aligned on the timeline. The data from different modality can be taken as different dimensions of multivariate time series. The *i*_th student sample’s feature data can be represented as:


$$S_{i}=(\begin{array}{cccc} E_{1} & E_{2} & \ldots & E_{\omega }\\ T_{1} & T_{2} & \ldots & T_{\omega }\\ Q_{1} & Q_{2} & \ldots & Q_{\omega } \end{array})$$

#### 3.2.3 Coarse-grained assessment based on deep learning.

At this stage, a student engagement recognition model is constructed to predict the level of engagement. In this work, engagement levels are categorized into three levels: high engagement, moderate engagement, and low engagement, providing a coarse-grained differentiation of student engagement states.

Multimodal data are collected during the learning process. Since, they can be considered a form of time series data. Discovering the interrelationships among different modal data and extracting valuable time-related information embedded in the data is important for SEA. Considering the temporal nature and multimodal characteristics of SEA data, this paper adopts a deep learning-based method for multimodal time series classification to address the recognition of engagement levels.

Among deep learning methods [39–41], deep Convolutional Neural Network (CNN) is the most widely applied architecture for time series processing [42]. Deep CNNs exhibit notable advantages in nonlinear modelling, automatic feature learning, which can meet the demand of processing time series data. However, the potential of deep CNNs to get engagement level have yet to be further explored. In this work, we examine four popular CNNs suitable for time series data. These four models are Time Convolutional Neural Network(Time-CNN), Fully Convolutional Neural Network(FCN) , Encoder and Multiscale Convolutional Neural Network (MCNN). These models have been demonstrated to achieve excellent classification performance on many time series datasets [42]. In the next section, we explore their applicability on collected educational dataset.

Time-CNN [43] is an optimization of traditional CNNs in terms of time series processing. There are three main differences compared to the traditional CNNs. The first is the use of the mean squared error (MSE) instead of the traditional categorical cross-entropy loss function. And, instead of a softmax classifier, the final layer is a traditional Fully Connected (FC) layer with sigmoid as the activation function, which does not guarantee a sum of probabilities equal to 1. Another difference to traditional CNNs is the use of a local average pooling operation instead of local max pooling. The network is composed of two consecutive convolutional layers with respectively 6 and 12 filters followed by a local average pooling operation of length 3. The convolutions adopt the sigmoid as the activation function. The network’s output consists of an FC layer with a number of neurons equal to the number of classes in the dataset.

FCN [44] for classifying time series is first composed of three convolutional blocks where each block contains three operations: a convolution followed by a batch normalization whose result is fed to a ReLU activation function. The result of the third convolutional block is averaged over the whole time dimension which corresponds to the Global Average Pooling (GAP) layer. Finally, a traditional softmax classifier is FC to the GAP layer’s output. All convolutions have a stride equal to 1 with a zero padding to preserve the ex-act length of the time series after the convolution. The first convolution contains 128 filters with a filter length equal to 8, followed by a second convolution of 256 filters with a filter length equal to 5 which in turn is fed to a third and final convolutional layer composed of 128 filters, each one with a length equal to 3. It does not contain any local pooling layers which means that the length of a time series is kept unchanged throughout the convolutions. In addition, the replacement of the traditional final FC layer with a Global Average Pooling (GAP) layer reduces drastically the number of parameters in a neural network.

Encoder [45] is a hybrid deep CNN whose architecture is inspired by FCN with a main difference where the GAP layer is replaced with an attention layer. Specifically, a channel-wise attention mechanism is employed. The feature map output from the convolutional layers is split into two parts: one part holds the feature data, while the other part is used to generate attention weights. The attention weights are computed using a softmax activation, which normalizes the weights across the channels, effectively assigning different levels of importance to each feature channel. These attention weights are then applied to the feature data through an element-wise multiplication operation, allowing the model to focus on the most relevant features for the task. This mechanism enhances the model’s ability to emphasize important channels, thereby improving performance on the classification task. Similarly to FCN, the first three layers are convolutional with some relatively small modifications. The first convolution is composed of 128 filters of length 5; the second convolution is composed of 256 filters of length 11; the third convolution is composed of 512 filters of length 21. Each convolution is followed by an instance normalization [46] operation whose output is fed to the Parametric Rectified Linear Unit (PReLU) [47] activation function. The output of PReLU is followed by a dropout operation (with a rate equal to 0.2) and a final max pooling of length 2. The third convolutional layer is fed to an attention mechanism [48] that enables the network to learn which parts of the time series (in the time domain) are important for a certain classification. Finally, a traditional softmax classifier is fully connected to the latter layer with a number of neurons equal to the number of classes in the dataset.

MCNN [49] consists of two convolutional layers followed by max pooling, an FC layer, and a softmax layer. However, it incorporates a complex data preprocessing step, including the Window Slicing (WS) method for data augmentation. This method involves sliding a window over the input time series to extract subsequences, which undergo transformations like identity mapping, down-sampling, and smoothing before feeding into independent convolutions. The output of each convolution is concatenated for subsequent layers. With 256 filters per convolution and sigmoid activation, the network utilizes max pooling and crossvalidates filter length and pooling factor hyperparameters. The WS method is also applied at test time for class determination via majority vote over predicted labels of extracted subsequences.

The trained model can be used to predict new student sample and provide probabilities for engagement levels. Accordingly, the highest probability value corresponds to the final predicted engagement level.

#### 3.2.4 Fine-grained assessment based on interpretability.

Coarse-grained assessment can capture students’ engagement level. However, it is difficult to distinguish the differences between students at the same level. In deep learning models, analyzing the gradient changes of feature data points allows us to quantify their impact on the final prediction decision. Leveraging the interpretability of deep learning models, we provide an engagement scoring method for further refinement of engagement levels, as detailed in Algorithm 1.

To understand the varying impact of each feature data point on the engagement level and to quantify a student’s engagement score, the following steps are typically taken:

Step 1: Gradient Magnitude Calculation

Feed the *i*_th student sample(*S*_*i*_) into the trained CNN model and record the model’s predictions.

Use the backpropagation to compute gradients with respect to the input sample, which will indicate how each feature contributes to the engagement level.

The gradient ${▽}_{S_{i}}ℒ$ is calculated as Eq 6.

$${▽}_{S_{i}}ℒ=\frac{\partial ℒ}{\partial S_{i}}$$ (6)

where ℒ is the loss function (cross-entropy loss) and (*S*_*i*_) is the student sample input. The cross-entropy loss is calculated as Eq 7.

$$ℒ=-\sum_{i=1}^{N}y_{i}log\left(\hat{y_{i}}\right)$$ (7)

where, *N* is the total number of classes, *y*_*i*_ is the actual class label. $\hat{y_{i}}$ is the predicted probability of class i by the model.

*S*_*i*_ refers to the feature data of the student sample, typically representing numerical values of the student’s engagement during learning. The gradient indicates how much each feature affects the model’s prediction. A larger gradient implies a greater influence of the feature on the prediction.

**Algorithm 1. Calculating student engagement score.**

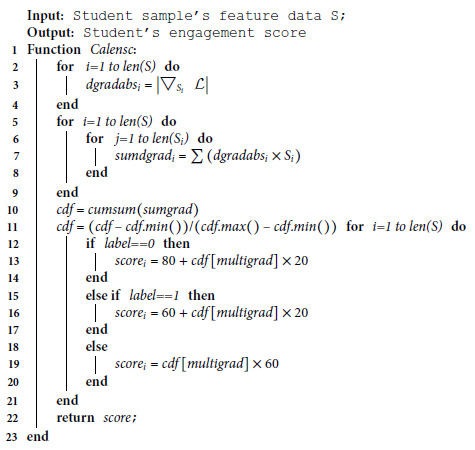



Step 2: Summing Gradient Contributions

After calculating the gradients, compute the magnitude of the gradients to measure their impact on the engagement level as Eq 8.

$$dgradabs_{i}=|{▽}_{S_{i}}ℒ|$$ (8)

where *dgradabs*_*i*_ represents the magnitude of the gradient for the *i*_th student sample. The total contribution value (*sumdgrad*_*i*_) is calculated as Eq 9.

$$sumdgrad_{i}=\sum (dgradabs_{i}\times S_{i})$$ (9)

For each student sample *S*_*i*_, the formula calculates the product of the absolute gradient and the corresponding feature data. This product reflects the extent to which the feature contributes to the engagement level.The formula uses a summation to calculate the total contribution of all features. For each student sample *S*_*i*_, the contributions of all features are summed up to get the total contribution value, which results in the overall engagement score for that sample.

Step 3: Engagement Quantization

To quantify the engagement, we utilize a segmented mapping strategy based on the Cumulative Distribution Function (CDF) to map the data into percentile values. The CDF method is widely used to capture the distribution of data points, and this approach helps to obtain intuitive engagement scores.

## 4 Data collection and annotation

We carried out blended teaching in "Computer Network" lab lesson for a duration of eight weeks. In the weekly lab lesson, the class begins with a 10 to 20 minutes of micro-lecture video learning, which cover relevant theoretical knowledge points and software operation demonstrations. During this period, students need to complete 1 to 2 tests, mainly simple multiple choice questions and fill-in-the-blank questions. The entire lesson is conducted via Tencent Meeting, allowing participating students to ask questions and engage in discussions through the chat box at any time. In the remaining time, students complete the experiment independently and are required to submit an experiment report. Students are required to have their webcams turned on during the sessions, with no other specific restrictions imposed. A total of 27 students are enrolled in this lesson. Among them, there are 12 females and 15 males, all from the same major: Information and Computing Science.

The engagement labels in this work are separated into three levels, including high engagement, moderate engagement, and low engagement. Three annotators determine the engagement level of the samples based on the strength and proportion of observed engagement cues (as shown in Table 2 ). Students with low engagement exhibit few signs of engagement, while Students with high engagement are actively involved throughout the learning process. Students with moderate engagement are able to participate in the learning process, but are susceptible to distractions. Representing a student’s data and labels for a weekly lesson as a single sample. Following data collection and annotation, we obtained a total of 205 valid samples, consisting of 23 with high engagement, 147 with moderate engagement, and 35 with low engagement. Following the fusion methods outlined in Sect 3, this forms an imbalanced multivariate time series dataset.

**Table 2:** Engagement cues of different engagement levels.

Cue	Student’s Performance
Low Engagement	No response to the teacher’s question.
	Displaying varied facial expressions, possibly including excitement, enthusiasm, dissatisfaction, and disappointment.
	Staring at the screen, leaning forward, and getting close to the screen.
	Leaving the seat.
	Napping.
Moderate Engagement	Responding to the teacher’s question.
	Facial expressions are relatively relaxed, with occasional smiles, furrowed brows, or other casual expressions.
	The mouth may open from time to time, possibly with speaking or chewing gum actions.
	The eyes exhibit more wandering gaze, potentially observing the surrounding environment or other objects.
	More bodily movements, such as swaying, fidgeting, or handling objects, and the use of a mobile phone may be involved.
High Engagement	Initiating questions or expressing opinions.
	A stable facial expression with a closed mouth, minimal and subtle body movements.
	Continuous focus on the screen with occasional slight eye wandering.

## 5 Results

All data are run on a computer with MacOS (Apple M2 Ultra chip), 24-core CPU (16 performance cores and 8 efficiency cores), 60-core GPU, a 32-core Neural Engine, and 128G memory. We use the Python programming language with keras to implement all the algorithms in experiments. Additional information abouthyperparameter settings for four CNNs can be found in Table 3.

**Table 3:** Hyperparameters for the deep learning models.

Models	Layers	Convs	Loss	Epochs
Time-CNN	3	2	MSE	2000
FCN	5	3	Entropy	2000
Encoder	5	3	Entropy	100
MCNN	4	2	Entropy	200

In order to verify the applicability and rationality of the proposed framework in real educational scenarios, a sufficient empirical comparative study is conducted. The study aims to answer the following research questions:

1. Can the proposed framework be effectively applied to real educational scenarios?
2. How applicable is the deep learning model? Which deep learning model can better distinguish different engagement levels?
3. Is the quantified engagement scores reasonable?

### 5.1 Model performance evaluation

The objective of the assessment is to classify the students correctly into their respective engagement levels. The common evaluation metric for such multiclassification tasks is Accuracy, Precision, Recall and F1-Score. These are computed individually for each of the levels. Taking into account the imbalance of the samples, we also use macro and weighted averages of these metrics to understand the overall CNNs performance.

The performance of four CNNs in Coarse-grained assessment is evaluated using these four metrics, Accuracy, Weighted Precision, Weighted Recall and Macro F1-Score. Table 4 reports the best results obtained after multiple runs. As shown in Table 4, Accuracy represents the proportion of correctly classified samples. FCN achieves the highest accuracy of 0.95, indicating its strong overall predictive capability. Encoder follows closely with an accuracy of 0.93. Time-CNN and MCNN have accuracies of 0.85 and 0.88, respectively, indicating relatively lower. Weighted Precision is the weighted average of precision for each class, considering class imbalances. FCN exhibits the highest weighted precision of 0.95, indicating its ability to correctly classify instances across different engagement levels. Time-CNN has the lowest weighted precisions of 0.75, respectively, suggesting some challenges in precision for certain engagement level. Weighted Recall is the weighted average of recall for each class, also considering class imbalances.

**Table 4:** Performance of four CNN models in coarse-grained learning investment evaluation.

Models	Time-CNN	FCN	Encoder	MCNN
Accuracy	0.85	0.95	0.93	0.88
Weighted Precision	0.75	0.95	0.93	0.90
Weighted Recall	0.85	0.95	0.93	0.88
Macro F1-score	0.58	0.91	0.88	0.85

All CNNs demonstrate high weighted recalls ranging from 0.85 to 0.95, indicating their ability to correctly identify relevant instances across classes while considering class weights. Macro F1-score is the average of the F1-scores for each class, treating each class equally, without considering class imbalances. FCN achieves the highest macro F1-score of 0.91, indicating a good balance between precision and recall across classes. Time-CNN has the lowest macro F1-scores of 0.58, respectively, suggesting variations in the balance between precision and recall for different classes or imbalanced class distributions.

Overall, FCN exhibits strong performance across multiple metrics, while Time-CNN may require further tuning or considerations for handling class imbalances or specific classrelated challenges. The performance of Encoder and MCNN slightly lags behind that of FCN.

**Fig 2:**
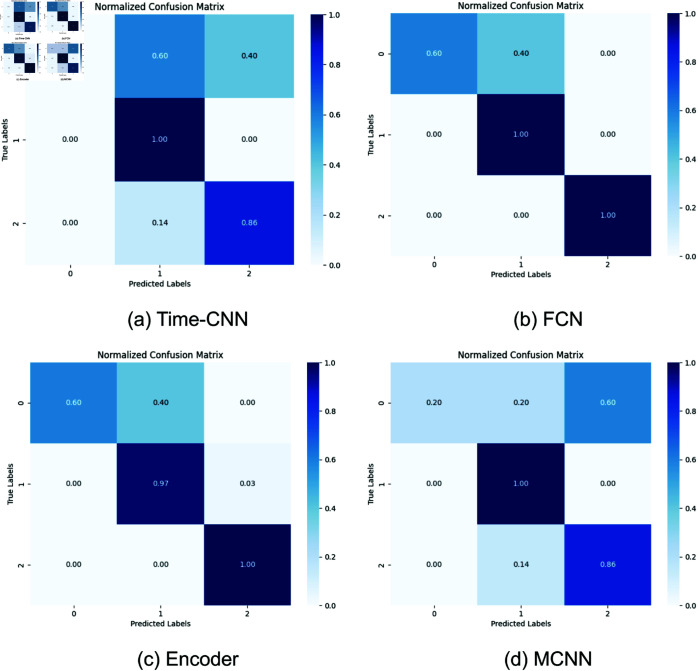
Normalized confusion matrix of four CNN models.

Fig 2 displays the normalized confusion matrices for four CNN models, where label 0 represents ’high engagement,’ label 1 represents ’ moderate engagement,’ and label 2 represents ’low engagement. Normalized confusion matrix is a representation of the confusion matrix in which the values in each row are normalized to show the proportion of true positive instances for the correct class and the proportion of false positive instances for each class. This normalization helps visualize the classifier’s performance in a more intuitive way, especially when dealing with imbalanced datasets. In a normalized confusion matrix. The diagonal elements represent the proportion of true positive instances (correctly classified) for each class. Off-diagonal elements represent the proportion of false positive instances (misclassified as another class) for each class.

The data in Table 3 indicates that FCN and Encoder have very similar performance metrics. However, from the normalized confusion matrix in Fig 2, a significant difference can be observed. FCN can correctly identify moderate engagement and low engagement, while Encoder can only correctly identify low engagement, with some classifications of moderate engagement. Time-CNN and MCNN exhibit similar performance, accurately identifying moderate engagement but performing poorly in recognizing low engagement. For high engagement, all models struggle to achieve satisfactory recognition.

From the above experimental results, it can be observed that deep learning model is effective in identifying the engagement level, with the FCN being particularly effective in distinguishing between different levels of engagement.

**Fig 3:**
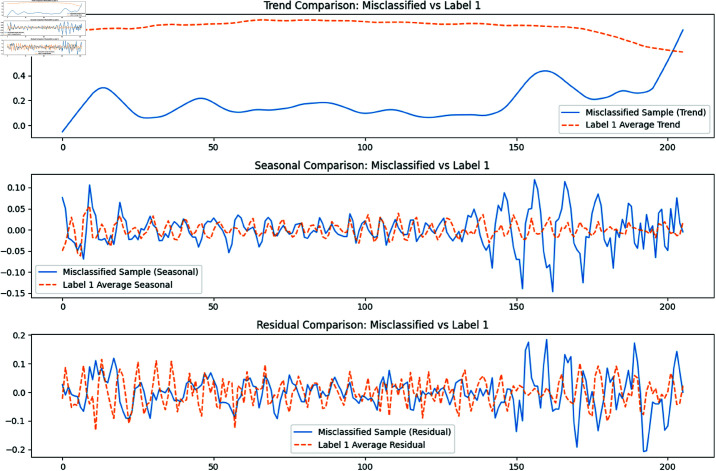
The STL decomposition for the TF-IDF sequence.

As shown in Fig 2, all four models show suboptimal performance in recognizing high engagement. The FCN model, which perform slightly better, can partially identify high engagement, but they are also prone to misclassifying it as moderate engagement. To investigate why the models misclassifies samples with label 0 as label 1, we use the Seasonal-Trend Decomposition using LOESS (STL) method to dissect the time series of both the misclassified samples and those labeled as 1 into their trend, seasonality, and residual components, subsequently analyzing their similarities. Our analysis indicates that, among the engagement feature sequences extracted across the three modalities, the TF-IDF sequence is the primary driver of these misclassifications. Fig 3 presents the visualization of the STL decomposition for the TF-IDF sequence, revealing that the average trend of the misclassified samples shares notable similarities with the average trend of label 1, particularly in its upward trajectory. However, the misclassified samples exhibit smaller fluctuations compared to the broader range observed in label 1’s trend. The seasonality also displays some resemblance in its periodic patterns between the two groups. In contrast, the residuals of the misclassified samples show significantly larger fluctuations, with amplitudes far exceeding those of the average residuals for label 1, suggesting that residual differences are unlikely to be the main cause of misclassification. Overall, the trends and seasonality of the misclassified samples exhibit partial overlap with those of label 1—representing moderate engagedment—especially in their shared rising trends and subtle periodic characteristics, which contribute to the model’s confusion.

### 5.2 Correlation analysis

Previous research has shown that student engagement is positively related to academic performance. In the experiment, based on this conclusion, the reasonableness of the quantified scores was validated. We regard the lab report scores submitted by students as academic performance, and analyze the correlation between the lab report scores and engagement scores.

**Fig 4:**
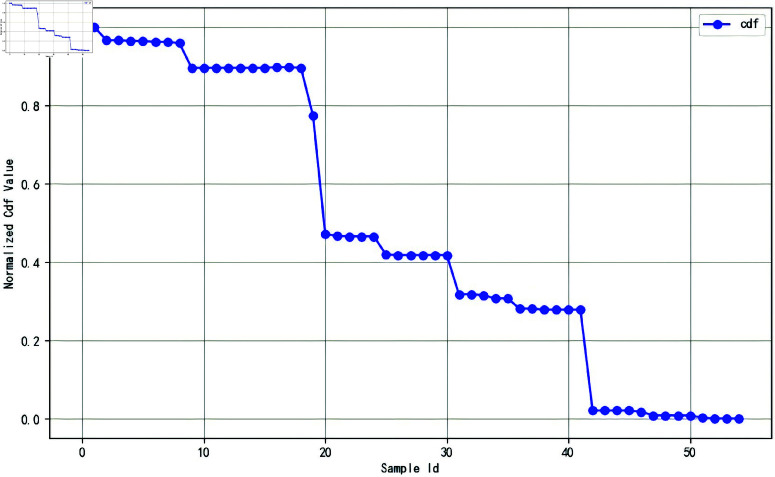
The normalized cdf value.

**Fig 5:**
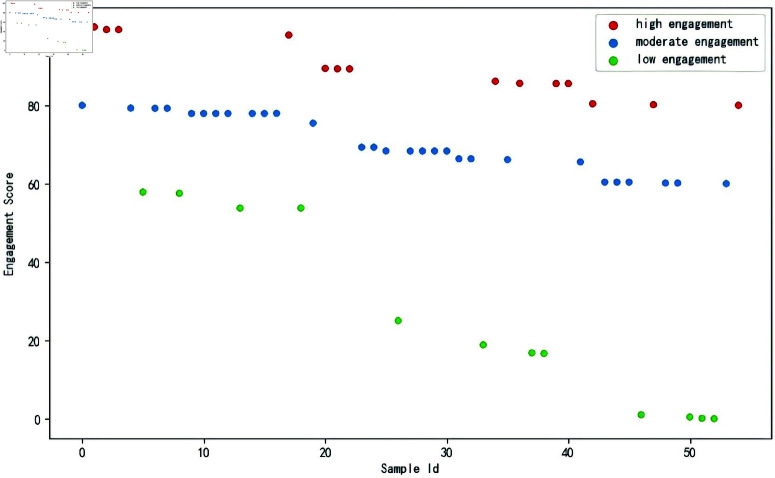
The engagement scores.

Following the method described in Algorithm 1, utilizing the gradient magnitude mapping of the trained FCN model, the engagement scores are computed. Fig 4 shows the normalized cdf value, and Fig 5 shows the engagement scores obtained by fine-grained assessment.

We employ the Spearman Rank Correlation Coefficient, a nonparametric statistical method, to measure the relationship between engagement scores and lab report scores. The Spearman correlation coefficient is used to assess the correlation between two sets of ranked or ordinal data without assuming a linear relationship between the variables. In the specific calculation process, engagement scores and experimental report scores are treated as pairs of data points. The values of each variable are ranked in ascending order, with the smallest value receiving a rank of 1, the second smallest a rank of 2, and so on. For each pair of data points, the squared difference in their ranks is computed, and the sum of these differences is calculated. This is done to measure the rank differences between the two variables. The formula for calculating the Spearman correlation coefficient is as Eq (10).

$$\rho =1-\frac{6\sum d_{i}^{2}}{n\left(n^{2}-1\right)}$$ (10)

The calculated Spearman correlation coefficient is ρ=0.8033543124054942.This indicates a strong positive correlation between the engagement scores and students’ academic performance. Consistent with previous research findings, this also indirectly confirms the reasonableness of fine-grained assessment. These scores are more intuitive than simple engagement levels and can differentiate differences within the same engagement level.

## 6 Conclusion

In this paper, we assess student engagement in a blended learning engagement scenario using a multimodal deep learning framework. The deep learning models demonstate strong capabilities in multimodal data fusion and time series data analysis. In terms of coarse-grained assessment, the applicability of four deep learning models, Time-CNN, FCN, Encoder and MCNN, to real educational datasets was explored. The experimental results show that the four deep learning modelseffectively identify students with moderate and low engagement, with the FCN performing the best. In terms of fine-grained assessment, a novel engagement score quantification method based on gradient magnitude mapping is proposed. Gradient magnitude mapping is a commonly used model interpretability technique in the field of image processing. It annotate the contribution of each data point in input sample to the engagement level. Therefore, the quantified engagement scores, based on the contribution of each data point, provide a more detailed refinement of the engagement level. After conducting a correlation analysis, we find that the quantified scores are positively correlated with academic performance, which aligns with previous research findings. This suggests that the score quantification method is reasonable and capable of further refining the distinctions within the same engagement level.

Future experiments will explore integrating additional modalities, such as physiological data and audio data, to enable the model to capture the more subtle aspects of student engagement. There are differences between short-term engagement and long-term sustained engagement. It is necessary to further enhance the ability to evaluate long-term engagement and its impact on students’ academic performance. Sustained engagement over time may differ from short-term engagement and could have different implications for learning performance. It is important to consider how to extend the framework to assess long-term engagement.

In deep learning, the accuracy of sample annotation has a significant impact on model training and performance. Multimodal data reflects subtle variations and differences in student engagement, which may not always be easy to categorize in educational environments. This, to some extent, impacts the scalability and deployment of frameworks in real-world settings. In future research, we will consider using fuzzy logic when classifying student engagement, integrating fuzzy methods into classification procedures to adapt to the dynamic needs of learning environments and improve uncertainty management and flexibility in assessing student engagement. Further exploreation of the application of other interpretable technologies will be considered. In addition to its application in quantifying engagement scores, consideration is also given to how to provide interpretable predictive explanations. This can help educators and students better understand the basis for the engagement scores.
